# Synthesis of middle–long–middle structured intralipids by biological catalysis and the evaluation of intralipids’ protective effect on liver injury rats

**DOI:** 10.1002/fsn3.2079

**Published:** 2021-03-23

**Authors:** Changsheng Liu, An’nan Chen, Li Xu, Tianqi Wang, Renwei Zhang, Juntao Xu, Yue Yu, Kaili Nie, Li Deng, Fang Wang

**Affiliations:** ^1^ Beijing Bioprocess Key Laboratory and State Key Laboratory of Chemical Resource Engineering College of Life Science and Technology Beijing University of Chemical Technology (BUCT) Beijing China; ^2^ Department of Hepatobiliary Surgery China‐Japan Friendship Hospital Beijing China; ^3^ National Research Institute for Family Planning Beijing China

**Keywords:** alcoholysis, esterification, intralipids, lipase, liver injury, MLM lipids

## Abstract

Intralipids are widely used to provide energy and necessary fatty acids for the patients. The structure of lipids may affect their function. We developed a bio‐catalyzed route to prepare various intralipids and investigated the protective effect of intralipids against α‐naphthylisothiocyanate (ANIT) induced liver injury rats, further discussing the structure–function relationship. The middle–long–middle (MLM) structural intralipid was synthesized through alcoholysis‐esterification, and the influence factors were investigated. ANIT treatment caused liver injury, further making hepatocyte damage, and increasing related biochemical indexes, like aspartate aminotransferase (AST), alanine transaminase (ALT), alkaline phosphatase (ALP), and total bilirubin (TBIL). Especially, MLM‐based and structoglyceride (STG) intralipids worked better in the early stage, to reduce the AST, ALT, and TBIL (*p* < .05). MLM showed a comparative advantage over other intralipids to accelerate the reduction of ALT (1st day) and AST (3rd day). MLM intralipid might be a promising next‐generation intralipid than the current STG intralipid liver‐injury patients. The biological catalysis MLM‐based intralipids can make the maximum utilization of fatty acids for the liver regeneration, where middle‐chain fatty acid (MCFA) in *sn*‐1,3 position can be metabolized directly to provide energy and long‐chain fatty acid (LCFA) in *sn*‐2 position can be delivered effectively for cell membrane repairing.

## Graphical abstract

Structural lipids were synthesized by the alcoholysis, esterification route. Through the lipids purification and high pressure homogenization, lipids were prepared as intralipids. Structural intralipids were found to improve the liver recovery in acute liver injury rats.

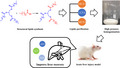

## 1 INTRODUCTION

Intralipid is wildly used in parenteral nutrition, providing energy and essential fatty acids for postoperative patients (Meguid et al., 1989). When the intralipid was injected into the vein, lipoprotein lipase was located in the endothelium, hydrolyzing triglyceride into *sn*‐2 monoglyceride and fatty acid, which would be absorbed and metabolized by the skeletal muscle, cardiac muscle, liver cells, and adipose cells(Nguyen et al., 2008). The metabolic absorption and endogenous synthesis of majority lipids occur in the liver (Huang et al., 2011). In the liver cell, middle‐chain fatty acid (MCFA) has a rapid energy‐supply rate, while long‐chain fatty acid (LCFA) could be used to synthesize endogenous triglycerides, phospholipids, and cholesterols (Nguyen et al., 2008).

**FIGURE 1:**
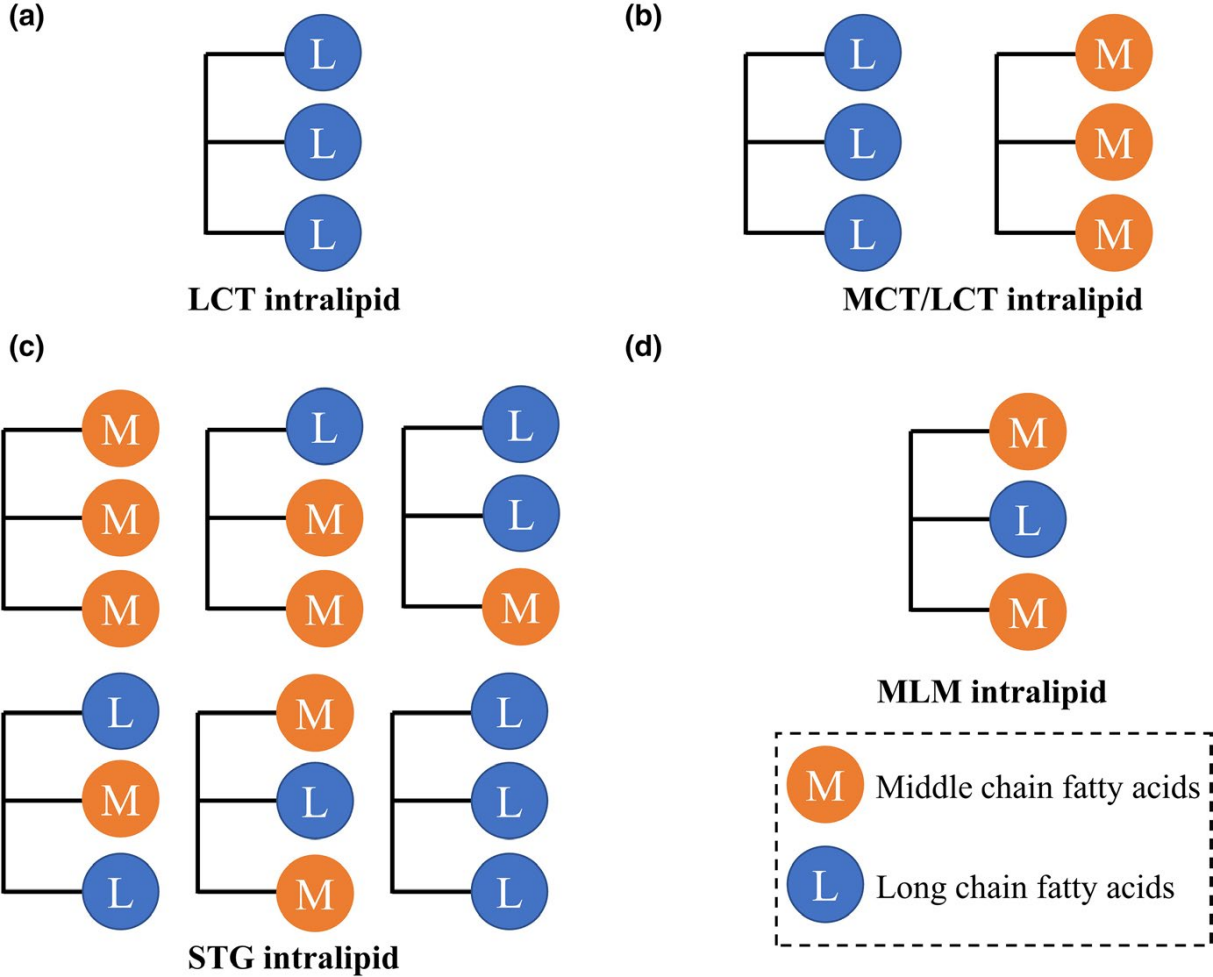
Lipids structure of different intralipids. (a) LCT intralipid; (b) MCT/LCT intralipid; (c) STG intralipid; (d) MLM intralipid

Postoperative patients with acute liver injury have a weak function of lipid metabolism in the liver (Reddy & Sambasiva Rao, 2006), who require specific intralipids to provide energy and essential fatty acids. Long‐chain triglyceride (LCT) intralipid with soybean oil as the oil phase (Wretlind, 1981) has a lower energy‐providing rate and metabolic rate, which cannot meet the immediate energy requirement of the patients (Figure 1a). With the addition of middle‐chain triglyceride (MCT), the MCT/LCT intralipid has a fast energy‐providing rate of MCT and can provide essential fatty acid of LCT to patients (Wicklmayr et al., 1988) (Figure 1b). However, the rapid metabolism of the MCT part causes the unstable energy supply and ketone body poisoning (Zhu & Li, 2013). To overcome this shortcoming, structoglyceride (STG) was developed by the hydrolyzation and random re‐esterification of MCT/LCT mixture (Figure 1c), while STG shows some advantages with stable energy supply and limited ketone body poisoning in postoperative patients (Min et al., 2012; Rubin et al., 2000; Zhu & Li, 2013). Compared with LCT and MCT/LCT intralipids, STG intralipids can maintain hepatic integrity and functions (Piper et al., 2008). Meanwhile, the lipid molecule structure in STG intralipid is nebulous, then, the hydrolyzed free LCFA may increase the burden of the lipid metabolism in acute injury liver. All the above intralipids mainly focus on the influence of fatty acids and rarely take the effect of fatty acids distribution on the glycerin skeleton into consideration. Middle–long–middle triglyceride (MLM) intralipid is the ideal lipid structure (Figure 1d), which can maximize the functional value of each fatty acid at the molecular level (Chambrier et al., 2006). The *sn*‐1,3 MCFA are available for immediate energy requirements and *sn*‐2 LCFA is available for functional requirements (Chambrier et al., 2006; Stein, 1999). The specific structure could meet the requirements of precise medicine and attract lots of interest. MLM intralipid was reported to have a faster lipid elimination rate in the healthy dog (Simoens et al., 2004). Thus, MLM intralipid may perform better for acute liver injury individuals, whose liver functions were impaired, and fat metabolisms were blocked.

The development of bio‐catalysis makes it possible to modify oils, like triglycerides (Devi et al., 2008) and phospholipid (Inoue et al., 2016). Lipases could place specific fatty acids in a certain position of glycerol bone, further maximizing the function of each specific fatty acids (Choi et al., 2012; Wei et al., 2019; Xu, 2000). In this paper, we designed and synthesized a specific MLM structured lipid through alcoholysis‐esterification strategy. The alpha‐naphthylisothiocyanate (ANIT)‐treated rat of acute liver injury was used to verify the protective function of various intralipids (MCT/LCT mixed triglyceride, random STG triglyceride, and MLM structural triglyceride). Through the analysis of the blood biochemical index, the effect of various intralipids on the recovery of liver function was evaluated.

## 2 MATERIALS AND METHODS

### 2.1 Materials

Caprylic acid (≥98%), and capric acid (≥98%) were of analytical grade (AG) and purchased from Xi Long Chemical Co. Ltd. Glycerol, H_2_SO_4_, NaOH, NaHCO_3_, Na_2_CO_3_, ethyl alcohol, and NaCl were of AG and purchased from Guang Fu Co. Ltd, Tianjin, China. Soybean oil was purchased from the local market. Soybean phospholipid (>90%) was purchased from Yuan Ye Bio‐Technology Co. Ltd, Shanghai, China. Novozym 435 (10,000 U/g, *Candida antarctic* lipase B), Lipozyme RM IM (275 U/g, lipase from *Rhizomucor miehei*), and Lipozyme TL IM (250 U/g, lipase from *Thermomyces lanuginosus*) were purchased from Novozymes. *Candida* sp. 99–125 lipase (8,000 U/g) was purchased from Beijing CAT New Century Biotechnology Co., Ltd. ANIT (α‐naphthylisothiocyanate) was purchased from Sigma Chemical Co., St. Louis, MO, USA. Hematoxylin and Eosin Staining Kit was purchased from Solarbio@ Life Sciences.

### 2.2 Synthesis of various triglycerides

#### 2.2.1 Synthesis of MCT

Two MCTs, including caprylic triglyceride and capric triglyceride, were prepared by chemical esterification with H_2_SO_4_ as catalysis, respectively. Caprylic acid was mixed with glycerol in a mole ratio of 1:3.5. The esterification reaction was performed under the condition of 500 bar, 100°C, with 1% H_2_SO_4_ as the catalyst. After 12 hr, the reaction mixture was collected and washed by a saturated NaCl solution for three‐time to remove the H_2_SO_4_. The extra caprylic acid was removed by alkali‐refining (Chumsantea et al., 2012). Caprylic triglyceride with a purity of 98% was obtained. Then, capric triglyceride (>96%) was synthesized under similar conditions.

#### 2.2.2 Synthesis of base lipids for intralipids

Three base lipids were synthesized, including MCT/LCT triglyceride, random STG triglyceride, and MLM structural triglyceride. MCT/LCT triglyceride was prepared by mixing 5 g MCT mixture (Caprylic triglyceride: capric triglyceride = 7:4, in mole ratio (Simoens et al., 2004; Wanten & Calder, 2007)) with 5 g LCT (soybean oil) directly. STG triglyceride, the random structural lipid, was synthesized by the transesterification of MCT and LCT. A mixture with the same amount of MCT and LCT in the above was prepared, where Novozym 435 was used for the random transesterification (Korma et al., 2018; Verdasco‐Martín et al., 2018). The mixture was incubated at 50°C for 12 hr, with Novozym 435 (10% wt to total oil) as catalyst (Korma et al., 2018). After the filtration of lipase, the random STG triglyceride (glyceride content > 99.1%) was obtained.

The synthesis of MLM structural triglyceride adopted the alcoholysis‐esterification strategy (Liu et al., 2020). First, soybean oil and ethanol were catalyzed to produce long‐chain *sn*‐2 monoacylglycerol (2‐MAG) by lipase (10% wt to soybean oil) (Liu et al., 2020). 2 g soybean oil (molar mass ≈ 880 g/mol^−1^), 0.2 g lipases, soybean oil: alcohol = 1:6–1:24 (mole ratio), 50 mg molecular sieves were placed in 25 ml conical flask with stopper, incubating at the condition of 20°C–50°C, and 200 rpm. Various factors that affect the synthesis of long‐chain 2‐MAG were investigated. The conversation of long‐chain 2‐MAG from TAG was measured by GC (Liu et al., 2020), followed the formula (1):

(1) $$\theta =\frac{\mathrm{MAG}}{\mathrm{MAG}+\frac{1}{2}\mathrm{DAG}+\frac{1}{3}\mathrm{TAG}}$$

Under the optimal conditions, a scale‐up alcoholysis was performed with 100 g soybean oil as substrates. At the end of alcoholysis, lipase and molecular sieves were removed by filtration. Then, the superfluous ethanol was evaporated by rotary evaporators at 600 pa, 80°C. Residues containing long‐chain 2‐MAG and fatty acid ethyl ester (FAEE) were separated by short path distillation, as described in our previous work (Liu et al., 2020). The separated long‐chain 2‐MAG was esterified with MCFA mixtures (Caprylic acid: capric acid = 7:4, in mole ratio) by Lipozyme TL IM, followed our previous reports (Liu et al., 2020). At the end of the reaction, lipases were removed by the method described above. Then, extra middle chain fatty acid mixtures were separated by short path distillation (Liu et al., 2020), and purified MLM structural lipids were obtained.

### 2.3 Preparation of intralipid

A high‐pressure homogenizer can be used to prepare intralipids by high‐speed impact and cavitation (Schuh et al., 2014). Based on the international requirement, the average particle size of intralipids should be lower than 500 nm, and the percentage of 5 μM particle should be lower than 0.05% v/v (Driscoll, 2006; Hippalgaonkar et al., 2010). Liquid with 10% wt MCT/LCT was used to optimize the processing conditions for high‐pressure homogenization. The preparation of intralipid referred to Alayoubi et al., (2015), including macro emulsion preparation, high‐pressure homogenization, membrane filtration, and high‐temperature sterilization.

### 2.4 ANIT‐treated model rat

40 Male 5‐week‐old SPS rats (180–220 g) were purchased from Beijing Laboratory Animal Research Center (BLARC). All the animals were treated under the “Guides for the Care and Use of Laboratory Animals” of the Committee of Animal Research, BLARC. Rats were housed in a controlled environment with a temperature of 21–26°C, the humidity of 40 ± 70%, and 12 hr light/12 hr dark cycle (Yamaura et al., 2012). Rats were acclimatized for 1 week and divided into five groups (control group, model group, MCT/LCT group, MLM group, and STG group).

Rats are free to water and food. Before ANIT oral administration, rates were fasting for 18 hr. Then, rats were orally administered a single loading dose of 100 mg/kg B.W. ANIT in olive oil (Chang et al., 2005). At 48 hr, injecting rats with various intralipids through the caudal vein (5 ml/kg). After the injection of intralipids, blood samples were taken from the caudal vein on 1st day, 3rd day, and 7th day. Blood samples were certificated by 1,600× *g* and stored under −20°C, for further analysis. *p < .05 represents a significant difference.

### 2.5 Analysis method

#### 2.5.1 Composition analysis of samples in GC

Free fatty acids (FFA), FAEE, monoacylglycerol (MAG), diacylglycerol (DAG), and triacylglycerol (TAG) were analyzed by gas chromatography (GC) with an FID detector and DB‐1 column. 10 μl samples are dissolved in 1 ml n‐hexane and 1 μl solution was injected. The temperature of the injection port was set as 360°C, and the FID detector was set as 380°C. The programming temperature of the column was as follows: the initial temperature was set at 200°C for 0.2 min, increased to 340°C at 8°C/min and maintained for 27.5 min (Liu et al., 2016).

#### 2.5.2 *sn*‐1,3 and sn‐2 fatty acids composition analysis

Gas chromatography equipped with FID detector and DB‐wax column, and the 37 mixed fatty acids methyl esters (FAMEs) standards were used to measure fatty acids in *sn‐1,3* and *sn‐2* (Liu et al., 2016). The analysis of total fatty acids composition was taken by the methyl esterification of samples and GC analysis with the DB‐wax column. Through the hydrolysis by porcine pancreatic lipase, samples containing fatty acids from the *sn*‐1,3 position, and MAG from the *sn*‐2 position. Then, samples were separated by silica gel G TLC plate with developing solvent of petroleum ether: ethyl ether: formic acid = 70:30:1 (v/v/v). The MAG band was scraped and methylated for GC analysis with the DB‐wax column. The temperature of the injection port and the FID detector were set as 220°C and 230°C, respectively. The temperature program of column was as follows: the initial temperature was set at 50°C for 1 min, increased to 150°C at 10°C/min and maintained for 2 min, then, increased to 220°C at 10°C/min and maintained for 25 min. The calculation of *sn‐1,3* and *sn‐2* fatty acids followed the reported method (Zhang et al., 2016).

#### 2.5.3 Blood biochemical index detection

The serum levels of aspartate aminotransferase (AST), alanine transaminase (ALT), alkaline phosphatase (ALP), and total bilirubin (TBIL) were analyzed by the Blood Biochemical Analyzer (OLYMPUS AU480, OLYMPUS, Japan) (Guo et al., 2019). Instrument working environment was as follows: temperature 18–32°C, and humidity 40 ± 80%. All the related reagents were provided by Intec PRODUCTS, INC. (China, Xiamen).

#### 2.5.4 Statistical analysis

Statistical analysis was performed by SPSS 19.0 and the significance of difference was tested by ANOVA. A *p*‐value < .05 was considered statistically significant.

## 3 RESULTS AND DISCUSSION

### 3.1 The optimization and preparation of MLM structural lipid

**FIGURE 2:**
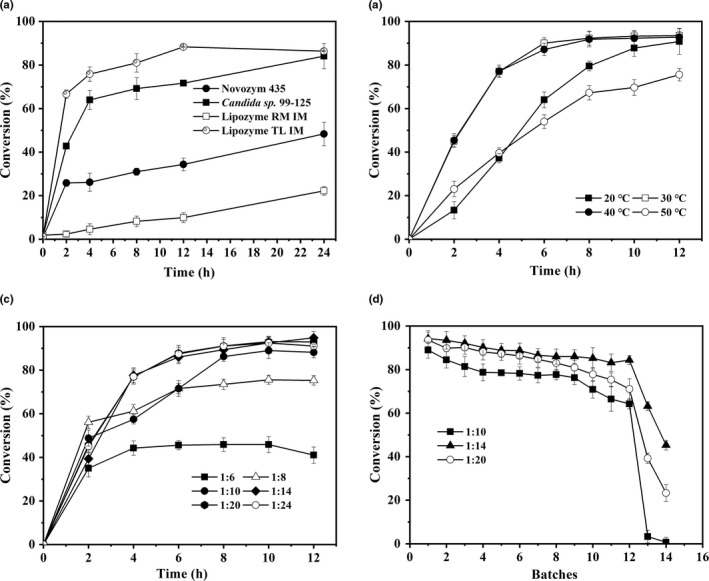
Effects of various factors on the conversation of 2‐MAG. (a) Lipases (10% wt to soybean oil, Novozym 435, Lipozyme RM IM, Lipozyme TL IM, *Candida* sp. 99–125), soybean oil: alcohol = 1:20, 45°C; (b) Temperature (20°C–50°C), Lipozyme TL IM (10% wt to soybean oil), soybean oil: alcohol = 1:20; (c) Substrate mole ratio (soybean oil: alcohol = 1:6–1:24 in mole ratio), Lipozyme TL IM (10% wt to soybean oil), 30°C; (d) Reusage time of enzymes. Substrate mole ratio (soybean oil: alcohol = 1:10–1:20 in mole ratio), Lipozyme TL IM (10% wt to soybean oil), 30°C, 12 hr per batch. All reactions were performed with 2 g soybean oil as substrates and 50 mg molecular sieves, at 200 rpm

The efficient conversation of 2‐MAG from TAG required 1,3‐regiospecificity lipases (Pfeffer et al., 2007). Four lipases, including Novozym 435, Lipozyme RM IM, Lipozyme TL IM, and *Candida* sp. 99–125 were adopted and compared. Among these four lipases, Lipozyme TL IM and *Candida* sp. 99–125 contributed the largest conversation of 2‐MAG, as high as 88.3% (12 hr) and 84.1% (24 hr) (Figure 2a), respectively. Taken the efficiency and reusability of lipase into consideration, immobilized lipase of Lipozyme TL IM was superior to the rest. Temperature is a key factor for enzymatic activities, which will influence the activity and lifetime of enzymes, as well as the mass transfer efficiency (Cao et al., 2017; Nie et al., 2006). In the alcoholysis of soybean oil, when the temperature was set at 30°C and 40°C, the conversion of 2‐MAG reached 93.5% and 92.8% at 12 hr, respectively (Figure 2b). To save the operation cost, 30°C was selected as the operating temperature. The effect of the substrate mole ratio on the alcoholysis was shown in Figure 2c, when the substrate mole ratio between soybean oil and alcohol varying from 1:10, 1:14, to 1:20, the conversion of 2‐MAG reached 88.2%, 94.4%, and 93.0%, respectively. Furthermore, batch tests under these different substrate ratios were investigated and demonstrated in Figure 2d. Over 94% conversion of 2‐MAG could be obtained with a mole ratio of 1:14, while the conversion of 2‐MAG was less than 80% after 12 batches in the rest. Thus, the optimal conditions of the alcoholysis of 2‐MAG from soybean oil were Lipozyme TL IM (10% wt to soybean oil), 30°C, and the substrate mole ratio of 1:14 (soybean oil: alcohol).

**FIGURE 3:**
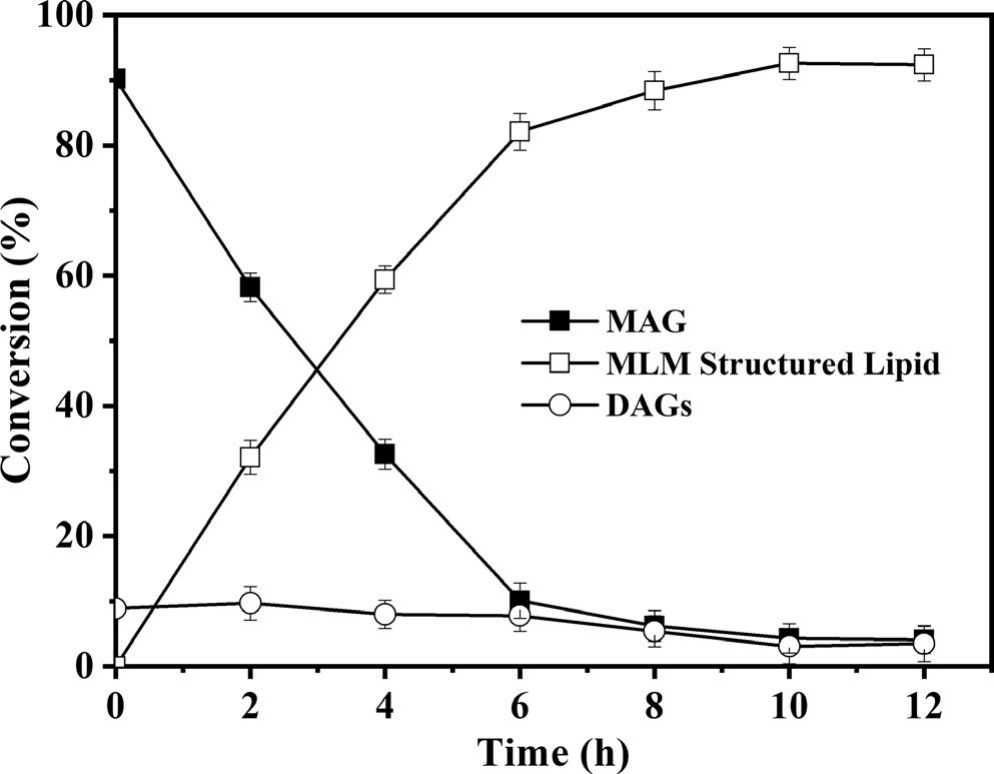
The time course of esterification between 2‐MAG and MCFAs. The esterification was performed with 20 g MAG, a mole ratio of MAG to MCFAs = 1:7 (caprylic acid: capric acid = 7:4, in mole ratio), Lipozyme TL IM (15 wt% by weight of MAGs), and nitrogen flow to remove generated water, at 45°C and 200 rpm

Short path distillation was used for the separation and purification of 2‐MAG from alcoholysis products (Liu et al., 2020). After the secondary short path distillation, the content of MAG reached 90.2% (Table S1), with 2.1% palmitic acid, 1.1% stearic acid, 25.6% oleic acid, 64.3% linoleic acid, and 6.9% linolenic acid. The esterification method was described in our previous work (Liu et al., 2020) and the composition of mixed middle fatty acid was caprylic: capric = 7:4, in mole ratio. The content of MLM structured lipid increased to 92.6% in 10 hr and fluctuated very slightly in the later (Figure 3). The reaction mixture was separated by the short path distillation with the evaporation wall of 120°C to remove middle chain fatty acid and the MLM structured lipid (≥90%) was obtained.

### 3.2 Fatty acids analysis for various lipids

Prepared MCT/LCT, STG, and MLM had a similar composition of total fatty acids, while the position of fatty acids in each lipid was different (Table 1). The total MCFA (C_8_–C_12_) and LCFA (C_16_–C_20_) in each lipid was around 50%. Although MCT/LCT and STG had similar fatty acid compositions in each position (total, *sn‐2*, and *sn‐1,3*), their molecule structures were different (shown in Figure S1), further leading to a different performance during metabolism. STG with randomly reassigned fatty acids on its glycerol backbone, bring a stable energy supply, limited ketone body poisoning, and positive nitrogen‐balance (Sadu Singh et al., 2020; Zhu & Li, 2013). Most of the LCFAs (90.58%) in MLM were concentrated in *sn*‐2, while the majority MCFAs (67.44%) located at *sn*‐1,3. This specific structure of MLM may contribute to a timely energy providing rate from MCFA in *sn‐1,3* and better delivery of LCFA in *sn‐2* (Chambrier et al., 2006). Hence, MLM may maximize the value of each fatty acids, where MCFA can be hydrolyzed for consumption as energy by β‐oxidation and LCFA can be delivered effectively as *sn‐2* MAG for intracellular construction (Chambrier et al., 2006; Stein, 1999).

**TABLE 1:** The fatty acid composition of different lipids expressed in wt% (MCT/LCT, MLM, and STG)^a^

	MCT/LCT			MLM			STG		
	Total	*sn*‐2	*sn*‐1,3	Total	*sn*‐2	*sn*‐1,3	Total	*sn*‐2	*sn*‐1,3
C8:0	29.06	27.68	29.75	28.52	6.74	39.41	29.50	31.62	28.44
C10:0	19.93	18.87	20.46	19.49	2.67	27.90	19.42	20.74	18.76
C12:0	0.22	0.18	0.24	0.09	0.01	0.13	0.11	0.15	0.09
C16:0	6.03	1.21	8.44	1.09	1.15	1.06	5.72	3.28	6.94
C18:0	1.75	0.57	2.34	0.98	1.76	0.59	2.05	1.21	2.47
C18:1	12.45	13.75	11.80	13.16	23.66	7.91	12.98	12.20	13.37
C18:2	27.34	34.06	23.98	33.26	57.82	20.98	27.11	27.67	26.83
C18:3	3.22	3.68	2.99	3.41	6.19	2.02	3.11	3.14	3.10
M	49.21	46.73	50.45	48.10	9.42	67.44	49.03	52.51	47.29
L	50.79	53.27	49.55	51.90	90.58	32.56	50.97	47.50	52.71

^a^ Results are the average of duplicate experiments.

### 3.3 Intralipids preparation and animal test results

#### 3.3.1 Intralipids preparation

The preparation of intralipids from lipids includes macro emulsion preparation, high‐pressure homogenization, membrane filtration, and high‐temperature sterilization, where the condition for high‐pressure homogenization was optimized (Table S2). The high‐pressure homogenization could homogenize various lipids very well, yielding 203.2 nm particles with 0.064 PdI. Membrane filtration and high‐temperature sterilization are used to sterilization, which would not break the particle size of intralipids (Table S3). In the end, 257.9 nm particles of intralipids were obtained with PdI of 0.103 and they met the related requirements (Driscoll, 2006; Hippalgaonkar et al., 2010).

#### 3.3.2 Blood biochemical analysis

**FIGURE 4:**
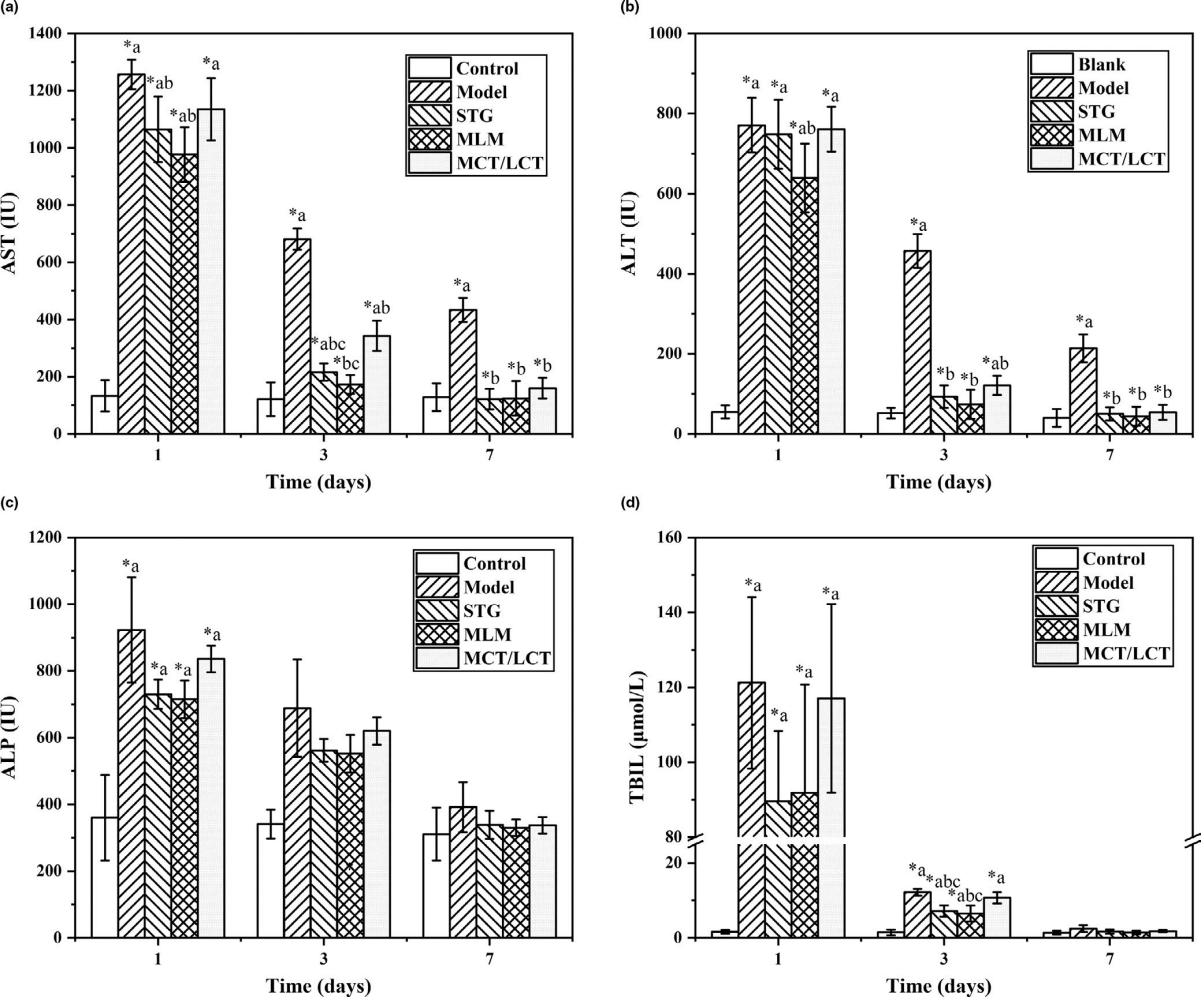
Effects of intralipid on serum biochemistry in ANIT‐induced cholestasis rats. ANIT‐induced liver injury rats (100 mg/kg ANIT) were treated with 5 ml/kg of STG intralipid, MLM intralipid, or MCT/LCT intralipid. The following liver function parameters were assayed: (a) aspartate aminotransferase (AST); (b) alanine aminotransferase (ALT); (c) alkaline phosphatase (ALP); (d) total bilirubin (TBIL). Data are expressed as the mean ± *SD* (*n* = 8 in each group). *^a^ *p* < .05 compared with the control group; *^b^ *p* < .05 compared with the ANIT‐inducted group (model); *^c^ *p* < 0.05 compared with the MCT/LCT group

Each group of intralipids contained eight rats, and no rats died in the experiment, indicating that prepared intralipids had a good safety and biocompatibility on rats. ALT, AST, and ALP are biochemical indexes that could reflect the level of hepatocyte injury directly, while TBIL measures hepatic secretory and excretory functions (Botsoglou et al., 2009; Zhang et al., 2018). ALT, AST and ALP are located in the liver cells. When the liver cells are damaged by ANIT, ALT, AST and ALP will leak from cells into the blood circulation, leading to the increase of their values. The dead red cell will produce bilirubin, which can be metablized by liver and excreted from biliary tract. Hence, the damaged liver will block this pathway and increase TBIL. ANIT treatment will cause an increase in ALT, AST, ALP, and TBIL (Yan et al., 2017). Compared with the control group, the ANIT‐inducted group had a significant increase in AST, ALT, ALP, and TBIL (*^a^ *P* < 0.05) on the first day, indicating a liver injury (Figure 4a–d). With time goes from 1 to 7 days, these biochemical indexes reduced with the liver recovered gradually. Interestingly, the treatment of intralipids could reduce AST, ALT, and TBIL significantly than the model group with *^b^ *P* < 0.05 in the early stage (Figure 4a,b,d), especially on the third day. However, intralipids did not lower the level of ALP significantly. This result supported that intralipids could accelerate liver recovery from injury.

On the first day, STG and MLM could reduce AST significantly (*^b^ *P* < 0.05), while MCT/LCT did not show a significant difference (Figure 4a). Especially, MLM intralipid could reduce the ALT of ANIT‐induced rats significantly in 1st day (*^b^ *P* < 0.05) (Figure 4b). On the third day, STG and MLM had a better treatment effect than MCT/LCT group in AST and TBIL with *^c^ *P* < 0.05 (Figure 4a,d). Interestingly, MLM worked better than STG in the treatment of AST, which reduced to the normal range of the control group (Figure 4a). On the seventh day, AST and ALT of the model group still had a significant increase than the control group with *^a^ *P* < 0.05 (Figure 4a,b). However, groups with the treatment of three intralipids had recovered to the normal range in AST, ALT, ALP, and TBIL.

We found that intralipids had the function of shortening the recovery time or treating the ANIT‐induced liver injury rats, especially in the reduction of AST, ALT, and TBIL. Our results were consistent with Ma et al., who reported that intralipids could improve the graft function and animal survival in liver transplant rats (Ma et al., 2007). The liver can regenerate rapidly from injury, meanwhile, it requires more phospholipid sources for the formation of the cell membrane and massive energy for cell replication (Michalopoulos, 2007). Intralipids could promote the regeneration process of the liver by providing necessary building materials for the formation of membranal phospholipid and energy for the liver regeneration by β‐oxidation. However, the structure of intralipids matters their promotion effect. MCT/LCT‐based intralipids showed a weak performance, because the hydrolyzed LCFA in *sn*‐1,3 position would increase the metabolic burden for these damaged liver cells. STG and MLM‐based intralipids worked better in the reduction of AST and ALT and TBIL than MCT/LCT intralipids, especially in the early stage. Some meta‐analysis researches have proved that the application of STG‐based intralipids could reduce AST (Li et al., 2019). The better promotion effect of STG intralipids is attributed to its random‐allocated fatty acids on glycerol backbone, which brings a more stable energy supply curve and positive nitrogen‐balance (Li et al., 2019; Ma et al., 2007). Interestingly, the MLM‐treated group showed a comparative advantage than others to accelerate the reduction of ALT (1st day) and AST (3rd day). The specific structure of MLM‐based intralipids placed different fatty acids on specific positions of triglyceride to deploy their best advantages. MCFAs were placed in *sn*‐1,3 position, which would be hydrolyzed to provide fast energy for liver regeneration (Karupaiah & Sundram, 2007; Sadu Singh et al., 2020). LCFAs were placed in the *sn*‐2 position, which will be absorbed in the form of monoglyceride form, and it can cross the cell membrane faster than the free form of LCFA (Bracco, 1994). Thus, LCFA was delivered effectively to provide precursors for intracellular construction, such as phospholipids in the cell membrane.

## 4 CONCLUSION

In this study, an enzymatic alcoholysis‐esterification of MLM structured lipid was investigated. The optimization of key factors involving lipases, temperatures, and substrate mole ratio was performed, to obtain a higher conversation of long‐chain 2‐MAG (over 94%). After esterification, MLM structured lipid (≥90%) was obtained. The distribution of fatty acids in triglycerides indicated that the MCFA is mainly located at *sn*‐1,3 position (67.44%) while LCFA is located at *sn*‐2 position (90.58%). The physiological function of the structure intralipids was evaluated by ANIT‐induced liver injury rats. MCT/LCT and STG with the similar fatty acid composition of MLM structured lipids were prepared, either.

In the treatment of ANIT‐induced liver injury rats, this paper found that various intralipid had the same product safety. These intralipids had similar fatty acids compositions, while their structures of lipid molecules were different. STG and MLM worked very well to reduce liver biochemical indexes in the early phase, like AST, ALT, and TBIL than MCT/LCT. Especially, MLM‐treated group showed a comparative advantage over other intralipids to accelerate the reduction of ALT (1st day) and AST (3rd day), proving that MLM intralipid might be a promising next‐generation intralipid than the current STG intralipid for liver‐injury patients. This paper lays a foundation for further research on structured intralipids. In the future, the detailed metabolism of intralipids with different structures should be done to investigate the mechanism.

## 5 STUDIES INVOLVING ANIMAL OR HUMAN SUBJECTS

All the animals were treated under the “Guides for the Care and Use of Laboratory Animals” of the Committee of Animal Research, BLARC.

## CONFLICTS OF INTEREST

The authors declared that they have no conflicts of interest in this work.

## Funding information

This work was supported by the National Key R & D Program of China (2017YFD0400603), National Natural Science Foundation of China (21978019, 21978020).

## Supporting information

Supinfo

## Data Availability

The data that support the findings of this study are available from the corresponding author upon reasonable request.
