# Supplementary material for: Synthesis of middle–long–middle structured intralipids by biological catalysis and the evaluation of intralipids’ protective effect on liver injury rats
Source: Food Sci Nutr. 2021 Mar 23;9(5):2381–9. doi: 10.1002/fsn3.2079 (PMC8116870; doi:10.1002/fsn3.2079)
Supplement: Supplementary file 1 — Supinfo [file FSN3-9-2381-s001.docx]

**Synthesis of middle-long-middle structured intralipids by biological catalysis and the evaluation of intralipids’ protective effect on liver injury rats**

**Short running title:** Structured intralipids in liver injury rats

Changsheng Liu^a^, An’nan Chen^a^, Li Xu^b^, Tianqi Wang^c^, Renwei Zhang^a^, Juntao Xu^a^, Yue Yu^a^, Kaili Nie^a*^, Li Deng^a*^, Fang Wang ^a^

^^[[1]](#footnote-1)^a^ Beijing Bioprocess Key Laboratory and State Key Laboratory of Chemical Resource Engineering, College of Life Science and Technology, Beijing University of Chemical Technology (BUCT), Beijing, 100029, PR China

^b^ Department of Hepatobiliary Surgery, China-Japan Friendship Hospital, Beijing, 100029, PR China

^c^ National Research Institute for Family Planning, Beijing 100081, PR China


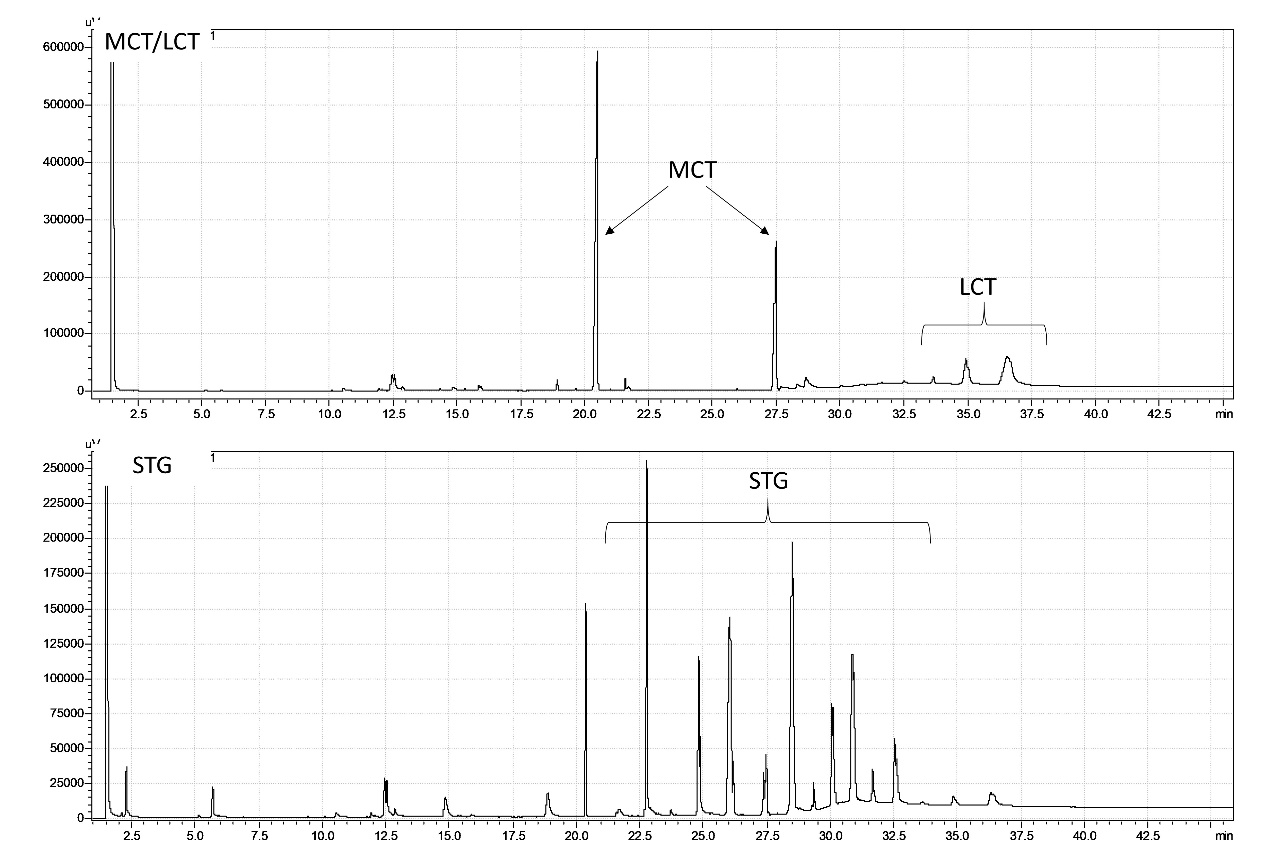


Figure S1: GC results of MCT/LCT and STG

Table S1: The Short path distillation procedure and the content percentage in each phase ^b^.

|  | Reaction mixture | Primary short path distillation | Secondary short path distillation |
| --- | --- | --- | --- |
| T_Evaporation wall_ (℃) | - | 120 | 150 |
| T_cooling wall_ (℃) | - | 45 | 45 |
| Scraper speed (rpm) | - | 250 | 250 |
| Pressure (Pa) | - | 0.1 | 0.1 |
| FFAE (%) | 74.2 | 9.8 | 0.9 |
| MAG (%) | 23.4 | 82.1 | 90.2 |
| DAG (%) | 2.4 | 8.1 | 8.9 |

^b^: Results are the average of duplicate experiments.

Table S2: The optimization for high pressure homogenization ^c^

| Content | Condition | Size (d. nm) | PdI |
| --- | --- | --- | --- |
| High pressure homogenization optimization | 300 bar, 3 rounds | 368.3 | 0.357 |
|  | 300 bar, 6 rounds | 397.7 | 0.349 |
|  | 300 bar, 10 rounds | 349.1 | 0.316 |
|  | 300 bar, 10 rounds+500 bar, 6 rounds | 203.2 | 0.064 |

^c^: Results are the average of duplicate experiments.

Table S3: The particle sizes of intralipids in different phase^d^

| Content | Condition | Size (d. nm) | PdI |
| --- | --- | --- | --- |
| Raw liquid | 500 rpm, 10 min | 965.1 | 1.000 |
| High pressure homogenization | 300 bar, 10 rounds+ 500 bar, 6 rounds | 203.2 | 0.064 |
| Membrane filtration | 0.45 μm Nylon membrane filtration | 200.9 | 0.102 |
| High temperature sterilization | 116°C, 30min | 257.9 | 0.103 |

^d^: Results are the average of duplicate experiments.

1. * Corresponding author Tel: +86-010-64414543; fax: +86-010-64416428.

   E-mail address: dengli@mail.buct.edu.cn (Li Deng).

   niekl@mail.buct.edu.cn (Kaili Nie). [↑](#footnote-ref-1)
